# (*E*)-1-(3-Bromo­phen­yl)-3-(3,4-dimeth­oxy­phen­yl)prop-2-en-1-one

**DOI:** 10.1107/S1600536812006836

**Published:** 2012-02-29

**Authors:** Carlos A. Escobar, Alexander Trujillo, Judith A. K. Howard, Mauricio Fuentealba

**Affiliations:** a Departamento de Ciencias Quimicas, Facultad de Ciencias Exactas, Universidad Andres Bello, Santiago, Chile; b Department of Chemistry, Durham University, Durham DH1 3LE, England

## Abstract

The mol­ecular structure of the title compound, C_17_H_15_BrO_3_, consists of a bromo­phenyl and a 3,4-dimeth­oxy­phenyl group linked through a prop-2-en-1-one spacer. The C=C double bond displays an *E* conformation, while the carbonyl group shows an *S*-*cis* conformation relative to the double bond.

## Related literature

For the Suzuki reaction, see: Miyaura & Suzuki (1995 ▶); Bringmann *et al.* (2005 ▶). For bichalcone derivatives, see: Shetonde *et al.* (2010 ▶). For related structures, see: Escobar *et al.* (2008 ▶); Valdebenito *et al.* (2010 ▶); Chu *et al.* (2004 ▶); Radha Krishna *et al.* (2005 ▶); Wu *et al.* (2005 ▶).
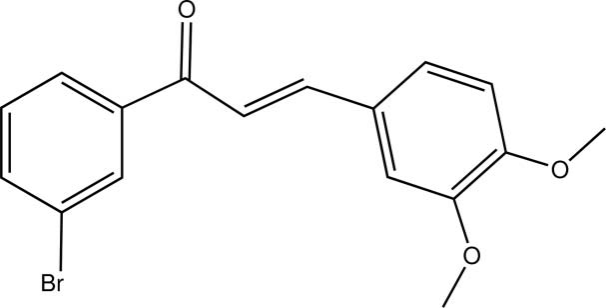


## Experimental

### 

#### Crystal data

C_17_H_15_BrO_3_
*M* *_r_* = 347.20
Monoclinic, 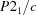

*a* = 12.7946 (5) Å
*b* = 3.9373 (1) Å
*c* = 29.8209 (10) Å
β = 109.219 (3)°
*V* = 1418.54 (8) Å^3^
*Z* = 4
Mo *K*α radiation
μ = 2.91 mm^−1^
*T* = 120 K
0.2 × 0.12 × 0.08 mm

#### Data collection

Agilent Xcalibur Sapphire3 Gemini ultra diffractometer
Absorption correction: multi-scan (*CrysAlis PRO*; Agilent, 2011 ▶) *T*_min_ = 0.802, *T*_max_ = 1.000
12861 measured reflections
3429 independent reflections
2895 reflections with *I* > 2σ(*I*)
*R*_int_ = 0.047

#### Refinement

*R*[*F* ^2^ > 2σ(*F* ^2^)] = 0.041
*wR*(*F* ^2^) = 0.074
*S* = 1.10
3429 reflections
192 parameters
H-atom parameters constrained
Δρ_max_ = 0.63 e Å^−3^
Δρ_min_ = −0.41 e Å^−3^

### 

Data collection: *CrysAlis PRO* (Agilent, 2011 ▶); cell refinement: *CrysAlis PRO*; data reduction: *CrysAlis PRO*; program(s) used to solve structure: *SHELXS97* (Sheldrick, 2008 ▶); program(s) used to refine structure: *SHELXL97* (Sheldrick, 2008 ▶); molecular graphics: *OLEX2* (Dolomanov *et al.*, 2009 ▶); software used to prepare material for publication: *OLEX2*.

## Supplementary Material

Crystal structure: contains datablock(s) global, I. DOI: 10.1107/S1600536812006836/fj2515sup1.cif

Structure factors: contains datablock(s) I. DOI: 10.1107/S1600536812006836/fj2515Isup2.hkl

Supplementary material file. DOI: 10.1107/S1600536812006836/fj2515Isup3.cdx

Supplementary material file. DOI: 10.1107/S1600536812006836/fj2515Isup4.cml

Additional supplementary materials:  crystallographic information; 3D view; checkCIF report

## Figures and Tables

**Fig. 1.**
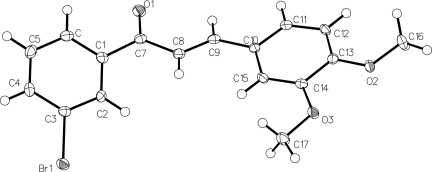
The molecular structure of title compound with full atom numbering scheme. Displacement ellipsoids are presented at 30% probability level and H atoms are shown as spheres.

## supplementary crystallographic information

### Comment

From the synthetic point of view, bromochalcones are the choice precursors to accomplish the C—C bond formation, through the Suzuki reaction, one of the most popular and powerful methods for coupling aryl–aryl moieties (Miyaura & Suzuki, 1995; Bringmann *et al.*, 2005), to produce symmetric or asymmetric biphenyls, this being the entry to bichalcones (Shetonde *et al.*, 2010).

The molecular structure of the title compound displays two phenyl rings connected through the organic prop-2-en-1-one spacer. As shown in Fig. 1, one phenyl ring is substituted at positions 3 and 4 with methoxy groups, while the other is substituted at position 3' with one Br atom.

The dihedral angle between the two aromatic rings joined by the conjugated spacer is 26.59 (9)°. On the other hand, the spacer formed by C10—C9—C8—C7—O1—C1 can be considered as a plane with a RMSD of 0.029 Å. This feature is also observed in other chalcones (Escobar *et al.* 2008; Valdebenito *et al.*, 2010; Chu *et al.* 2004; Radha Krishna *et al.* 2005; Wu *et al.* 2005).

Finally, both inter- and intramolecular hydrogen bonds are not observed in the crystalline packing of title compound.

### Experimental

A mixture of 3-bromoacetophenone (0.5 g, 2,5 mmol) and 3,4-dimethoxibenzaldehyde (0.41 g, 2,5 mmol), were dissolved in Methanol (50 ml), and were treated with KOH (2 g, dissolved in 20 ml methanol). After 20 min 30 ml of water were added, and the title compound precipitated as a yellow solid. Then, it was filtered and recrystallized in ethanol to yield 1.27 g (73%) of a yellow solid. m.p.117–120°C. IR (KBr): ν = 1657 (CO), cm^-1^. ^1^H-NMR (400 MHz, CDCl_3_): d = 3.94 (3*H*, s, OCH_3_), 3.96 (3*H*, s, OCH_3_), 6.90 (1*H*, d, *J* = 8.3 Hz, H5), 7.16 (1*H*, m, H2), 7.24 (1*H*, d, *J* = 7.2 Hz., H6), 7.32 (1*H*, d, *J* = 15.6 Hz., Ha), 7.38 (1*H*, t, *J* = 7.8 Hz., H5), 7.70 (1*H*, d, *J* = 7.9 Hz., H4), 7.77 (1*H*, d, *J* = 15.6 Hz., Hb), 7.93 (1*H*, d, *J* = 7.7 Hz., H6), 8.13 (1*H*, m, H2). ^13^C-NMR (400 MHz, CDCl_3_): d = 56.1, 110.2, 111.2, 119.4, 122.9, 123.5, 127.0, 127.6, 130.2, 131.4, 135.4, 140.4, 146.0, 149.3, 151.8, 189.1. HRMS calc. for C_17_H_15_BrO_3_ 346.02046; Found 346.019994.

### Refinement

The H atoms positions were calculated after each cycle of refinement using a riding model with C—H distances in the range 0.95—0.98 Å and *U*_iso_(H) = 1.2–1.5U_eq_(C).

### Crystal data

**Table:**

C_17_H_15_BrO_3_	*F*(000) = 704
*M**_r_* = 347.20	*D*_x_ = 1.626 Mg m^−^^3^
Monoclinic, *P*2_1_/*c*	Mo *K*α radiation, λ = 0.7107 Å
*a* = 12.7946 (5) Å	Cell parameters from 3587 reflections
*b* = 3.9373 (1) Å	θ = 2.6–29.0°
*c* = 29.8209 (10) Å	µ = 2.91 mm^−^^1^
β = 109.219 (3)°	*T* = 120 K
*V* = 1418.54 (8) Å^3^	Prism, colourless
*Z* = 4	0.2 × 0.12 × 0.08 mm

### Data collection

**Table:**

Agilent Xcalibur Sapphire3 Gemini ultra diffractometer	3429 independent reflections
Radiation source: Enhance (Mo) X-ray source	2895 reflections with *I* > 2σ(*I*)
Graphite monochromator	*R*_int_ = 0.047
Detector resolution: 16.1511 pixels mm^-1^	θ_max_ = 29.0°, θ_min_ = 2.6°
ω scans	*h* = −17→16
Absorption correction: multi-scan (*CrysAlis PRO*; Agilent, 2011)	*k* = −5→5
*T*_min_ = 0.802, *T*_max_ = 1.000	*l* = −40→40
12861 measured reflections	

### Refinement

**Table:**

Refinement on *F*^2^	Primary atom site location: structure-invariant direct methods
Least-squares matrix: full	Secondary atom site location: difference Fourier map
*R*[*F*^2^ > 2σ(*F*^2^)] = 0.041	Hydrogen site location: inferred from neighbouring sites
*wR*(*F*^2^) = 0.074	H-atom parameters constrained
*S* = 1.10	*w* = 1/[σ^2^(*F*_o_^2^) + (0.019*P*)^2^ + 1.5139*P*] where *P* = (*F*_o_^2^ + 2*F*_c_^2^)/3
3429 reflections	(Δ/σ)_max_ = 0.002
192 parameters	Δρ_max_ = 0.63 e Å^−^^3^
0 restraints	Δρ_min_ = −0.41 e Å^−^^3^

### Special details

**Table:**

Experimental. Empirical absorption correction using spherical harmonics, implemented in SCALE3 ABSPACK scaling algorithm.
Geometry. All e.s.d.'s (except the e.s.d. in the dihedral angle between two l.s. planes) are estimated using the full covariance matrix. The cell e.s.d.'s are taken into account individually in the estimation of e.s.d.'s in distances, angles and torsion angles; correlations between e.s.d.'s in cell parameters are only used when they are defined by crystal symmetry. An approximate (isotropic) treatment of cell e.s.d.'s is used for estimating e.s.d.'s involving l.s. planes.
Refinement. Refinement of *F*^2^ against ALL reflections. The weighted *R*-factor *wR* and goodness of fit *S* are based on *F*^2^, conventional *R*-factors *R* are based on *F*, with *F* set to zero for negative *F*^2^. The threshold expression of *F*^2^ > σ(*F*^2^) is used only for calculating *R*-factors(gt) *etc*. and is not relevant to the choice of reflections for refinement. *R*-factors based on *F*^2^ are statistically about twice as large as those based on *F*, and *R*-factors based on ALL data will be even larger.

### Fractional atomic coordinates and isotropic or equivalent isotropic displacement parameters (Å^2^)

**Table:**

	*x*	*y*	*z*	*U*_iso_*/*U*_eq_	
Br1	−0.11568 (2)	0.90741 (7)	0.528213 (9)	0.01853 (9)	
O1	−0.25274 (14)	0.3062 (5)	0.31571 (6)	0.0198 (4)	
O2	0.40922 (14)	0.3072 (5)	0.32327 (6)	0.0173 (4)	
O3	0.37580 (14)	0.5457 (5)	0.39741 (6)	0.0190 (4)	
C16	0.4322 (2)	0.1612 (7)	0.28340 (10)	0.0205 (6)	
H16A	0.3873	0.2751	0.2542	0.031*	
H16B	0.4141	−0.0814	0.2813	0.031*	
H16C	0.5109	0.1902	0.2874	0.031*	
C9	−0.0247 (2)	0.2800 (7)	0.32953 (9)	0.0143 (5)	
H9	−0.0759	0.1507	0.3052	0.017*	
C11	0.1119 (2)	0.1273 (6)	0.29162 (9)	0.0143 (5)	
H11	0.0535	0.0184	0.2676	0.017*	
C12	0.2171 (2)	0.1330 (6)	0.28749 (9)	0.0148 (5)	
H12	0.2298	0.0357	0.2606	0.018*	
C14	0.2834 (2)	0.4212 (7)	0.36346 (9)	0.0143 (5)	
C1	−0.2297 (2)	0.6078 (7)	0.38681 (9)	0.0140 (5)	
C15	0.1780 (2)	0.4234 (7)	0.36627 (9)	0.0144 (5)	
H15	0.1649	0.5243	0.3928	0.017*	
C5	−0.3827 (2)	0.8723 (7)	0.40364 (10)	0.0201 (6)	
H5	−0.4579	0.9429	0.3932	0.024*	
C10	0.0897 (2)	0.2760 (6)	0.32972 (9)	0.0130 (5)	
C3	−0.2072 (2)	0.8273 (6)	0.46427 (9)	0.0142 (5)	
C4	−0.3163 (2)	0.9344 (7)	0.45004 (10)	0.0178 (6)	
H4	−0.3450	1.0479	0.4716	0.021*	
C8	−0.0668 (2)	0.4429 (7)	0.35928 (9)	0.0149 (5)	
H8	−0.0187	0.5619	0.3858	0.018*	
C2	−0.1622 (2)	0.6663 (6)	0.43363 (9)	0.0135 (5)	
H2	−0.0869	0.5968	0.4442	0.016*	
C13	0.3031 (2)	0.2829 (6)	0.32325 (9)	0.0138 (5)	
C7	−0.1871 (2)	0.4390 (7)	0.35127 (9)	0.0143 (5)	
C17	0.3612 (2)	0.6874 (7)	0.43882 (9)	0.0212 (6)	
H17A	0.3096	0.8790	0.4298	0.032*	
H17B	0.4327	0.7667	0.4604	0.032*	
H17C	0.3313	0.5140	0.4548	0.032*	
C6	−0.3401 (2)	0.7083 (7)	0.37247 (10)	0.0175 (6)	
H6	−0.3866	0.6638	0.3409	0.021*	

### Atomic displacement parameters (Å^2^)

**Table:**

	*U*^11^	*U*^22^	*U*^33^	*U*^12^	*U*^13^	*U*^23^
Br1	0.02037 (14)	0.02143 (14)	0.01387 (14)	−0.00093 (12)	0.00574 (10)	−0.00270 (12)
O1	0.0164 (9)	0.0267 (11)	0.0153 (10)	−0.0041 (8)	0.0037 (8)	−0.0030 (8)
O2	0.0138 (9)	0.0241 (10)	0.0154 (10)	0.0002 (8)	0.0068 (7)	−0.0033 (8)
O3	0.0141 (9)	0.0302 (11)	0.0126 (10)	−0.0035 (8)	0.0042 (7)	−0.0081 (9)
C16	0.0222 (14)	0.0244 (15)	0.0198 (15)	0.0009 (12)	0.0138 (12)	−0.0032 (12)
C9	0.0151 (13)	0.0143 (12)	0.0115 (13)	−0.0014 (11)	0.0017 (10)	0.0032 (11)
C11	0.0151 (12)	0.0144 (13)	0.0115 (13)	0.0002 (10)	0.0018 (10)	0.0005 (11)
C12	0.0198 (13)	0.0146 (13)	0.0115 (13)	0.0031 (11)	0.0073 (10)	−0.0011 (11)
C14	0.0173 (12)	0.0132 (12)	0.0113 (13)	−0.0004 (11)	0.0030 (10)	−0.0002 (11)
C1	0.0150 (12)	0.0128 (12)	0.0148 (13)	−0.0034 (11)	0.0058 (10)	0.0026 (11)
C15	0.0190 (13)	0.0143 (12)	0.0111 (13)	0.0019 (11)	0.0065 (10)	−0.0001 (11)
C5	0.0137 (13)	0.0235 (15)	0.0237 (15)	0.0011 (11)	0.0071 (11)	0.0050 (13)
C10	0.0150 (12)	0.0115 (12)	0.0126 (13)	0.0025 (10)	0.0048 (10)	0.0031 (10)
C3	0.0156 (12)	0.0133 (13)	0.0131 (14)	−0.0031 (10)	0.0039 (10)	0.0019 (10)
C4	0.0168 (13)	0.0176 (13)	0.0217 (15)	0.0010 (11)	0.0102 (11)	−0.0001 (12)
C8	0.0146 (12)	0.0164 (13)	0.0128 (13)	−0.0009 (11)	0.0033 (10)	0.0017 (11)
C2	0.0104 (12)	0.0140 (13)	0.0174 (14)	−0.0024 (10)	0.0064 (10)	0.0030 (11)
C13	0.0141 (12)	0.0135 (12)	0.0141 (13)	0.0016 (10)	0.0052 (10)	0.0031 (11)
C7	0.0155 (12)	0.0124 (12)	0.0141 (13)	−0.0005 (11)	0.0036 (10)	0.0036 (11)
C17	0.0201 (14)	0.0285 (16)	0.0147 (14)	−0.0036 (12)	0.0054 (11)	−0.0065 (12)
C6	0.0159 (13)	0.0203 (14)	0.0141 (14)	−0.0026 (11)	0.0019 (10)	0.0025 (11)

### Geometric parameters (Å, º)

**Table:**

Br1—C3	1.907 (3)	C1—C2	1.398 (3)
O1—C7	1.232 (3)	C1—C7	1.498 (4)
O2—C16	1.436 (3)	C1—C6	1.391 (4)
O2—C13	1.361 (3)	C15—H15	0.9500
O3—C14	1.369 (3)	C15—C10	1.411 (3)
O3—C17	1.422 (3)	C5—H5	0.9500
C16—H16A	0.9800	C5—C4	1.387 (4)
C16—H16B	0.9800	C5—C6	1.383 (4)
C16—H16C	0.9800	C3—C4	1.384 (4)
C9—H9	0.9500	C3—C2	1.384 (4)
C9—C10	1.462 (3)	C4—H4	0.9500
C9—C8	1.343 (4)	C8—H8	0.9500
C11—H11	0.9500	C8—C7	1.477 (3)
C11—C12	1.391 (3)	C2—H2	0.9500
C11—C10	1.388 (3)	C17—H17A	0.9800
C12—H12	0.9500	C17—H17B	0.9800
C12—C13	1.387 (3)	C17—H17C	0.9800
C14—C15	1.379 (3)	C6—H6	0.9500
C14—C13	1.413 (4)		
			
C13—O2—C16	116.6 (2)	C11—C10—C15	118.5 (2)
C14—O3—C17	117.0 (2)	C15—C10—C9	123.0 (2)
O2—C16—H16A	109.5	C4—C3—Br1	118.8 (2)
O2—C16—H16B	109.5	C2—C3—Br1	118.96 (19)
O2—C16—H16C	109.5	C2—C3—C4	122.3 (2)
H16A—C16—H16B	109.5	C5—C4—H4	120.7
H16A—C16—H16C	109.5	C3—C4—C5	118.5 (2)
H16B—C16—H16C	109.5	C3—C4—H4	120.7
C10—C9—H9	115.8	C9—C8—H8	119.6
C8—C9—H9	115.8	C9—C8—C7	120.8 (2)
C8—C9—C10	128.5 (2)	C7—C8—H8	119.6
C12—C11—H11	119.0	C1—C2—H2	120.7
C10—C11—H11	119.0	C3—C2—C1	118.7 (2)
C10—C11—C12	122.0 (2)	C3—C2—H2	120.7
C11—C12—H12	120.5	O2—C13—C12	124.7 (2)
C13—C12—C11	119.0 (2)	O2—C13—C14	115.4 (2)
C13—C12—H12	120.5	C12—C13—C14	119.9 (2)
O3—C14—C15	125.2 (2)	O1—C7—C1	119.5 (2)
O3—C14—C13	114.5 (2)	O1—C7—C8	121.5 (2)
C15—C14—C13	120.3 (2)	C8—C7—C1	118.9 (2)
C2—C1—C7	122.0 (2)	O3—C17—H17A	109.5
C6—C1—C2	119.4 (2)	O3—C17—H17B	109.5
C6—C1—C7	118.6 (2)	O3—C17—H17C	109.5
C14—C15—H15	119.9	H17A—C17—H17B	109.5
C14—C15—C10	120.1 (2)	H17A—C17—H17C	109.5
C10—C15—H15	119.9	H17B—C17—H17C	109.5
C4—C5—H5	119.8	C1—C6—H6	119.6
C6—C5—H5	119.8	C5—C6—C1	120.7 (2)
C6—C5—C4	120.3 (2)	C5—C6—H6	119.6
C11—C10—C9	118.4 (2)		
			
Br1—C3—C4—C5	−179.3 (2)	C10—C11—C12—C13	2.0 (4)
Br1—C3—C2—C1	179.90 (18)	C4—C5—C6—C1	−1.0 (4)
O3—C14—C15—C10	−177.6 (2)	C4—C3—C2—C1	−0.5 (4)
O3—C14—C13—O2	−2.7 (3)	C8—C9—C10—C11	−171.3 (3)
O3—C14—C13—C12	176.7 (2)	C8—C9—C10—C15	7.1 (4)
C16—O2—C13—C12	−0.9 (4)	C2—C1—C7—O1	159.4 (2)
C16—O2—C13—C14	178.5 (2)	C2—C1—C7—C8	−22.0 (4)
C9—C8—C7—O1	−4.7 (4)	C2—C1—C6—C5	1.6 (4)
C9—C8—C7—C1	176.7 (2)	C2—C3—C4—C5	1.1 (4)
C11—C12—C13—O2	−179.5 (2)	C13—C14—C15—C10	2.3 (4)
C11—C12—C13—C14	1.1 (4)	C7—C1—C2—C3	179.2 (2)
C12—C11—C10—C9	175.6 (2)	C7—C1—C6—C5	−178.5 (2)
C12—C11—C10—C15	−2.9 (4)	C17—O3—C14—C15	0.2 (4)
C14—C15—C10—C9	−177.7 (2)	C17—O3—C14—C13	−179.8 (2)
C14—C15—C10—C11	0.7 (4)	C6—C1—C2—C3	−0.8 (4)
C15—C14—C13—O2	177.3 (2)	C6—C1—C7—O1	−20.6 (4)
C15—C14—C13—C12	−3.3 (4)	C6—C1—C7—C8	158.1 (2)
C10—C9—C8—C7	175.1 (2)	C6—C5—C4—C3	−0.3 (4)
